# Daytime-Dependent Changes of Cannabinoid Receptor Type 1 and Type 2 Expression in Rat Liver

**DOI:** 10.3390/ijms18091844

**Published:** 2017-08-24

**Authors:** Ivonne Bazwinsky-Wutschke, Alexander Zipprich, Faramarz Dehghani

**Affiliations:** 1 Department of Anatomy and Cell Biology, Martin Luther University Halle-Wittenberg, Grosse Steinstrasse 52, D-06108 Halle (Saale), Germany; faramarz.dehghani@medizin.uni-halle.de; 2 Laboratory of Molecular Hepatology, Clinic of Internal Medicine I, Martin Luther University Halle-Wittenberg, Ernst-Grube-Strasse 40, D-06120 Halle (Saale), Germany; alexander.zipprich@uk-halle.de

**Keywords:** endocannabinoid system, diabetes, diurnal rhythmic, hepatic cannabinoid receptors, streptozotocin, insulin, aging

## Abstract

The present study was performed to investigate the diurnal expression pattern of cannabinoid receptor type 1 (CB_1_) and type 2 (CB_2_) in liver tissue of 12- and 51-week-old normoglycemic Wistar rats. By using real-time RT-PCR, daytime dependent changes in both age groups and, for both, hepatic *Cnr1* and *Cnr2* receptor mRNA levels were measured. Highest amount of mRNA was detected in the light period (ZT3, ZT6, and ZT9) while the lowest amount was measured in the dark period (ZT18 and ZT21). Diurnal transcript expression pattern was accompanied by comparable changes of protein level for CB_1_, as shown by Western blotting. The current results support the conclusion that expression pattern of cannabinoid receptors are influenced by light/dark cycle and therefore seems to be under the control of a diurnal rhythm. These findings might explain the differences in the efficacy of cannabinoid receptor agonists or antagonists. In addition, investigation of liver of streptozotocin (STZ)-treated 12- and 51-week-old rats show alterations in the diurnal profile of both receptors *Cnr1* and *Cnr2* compared to that of normoglycemic Wistar rats. This suggests an influence of diabetic state on diurnal expression levels of cannabinoid receptors.

## 1. Introduction

Endocannabinoids belong to endogenous lipid ligands; among them, arachidonoylethanolamide (anandamide, AEA) and 2-arachidonoylglycerol (2-AG) are the most intensively studied [1]. Endocannabinoids exert their effects via cannabinoid receptor type 1 CB_1_ [2] and type 2 (CB_2_) [3]. These receptors are part of the family of G-protein coupled receptors with seven-transmembrane-domain.

CB_1_ was initially termed as the brain cannabinoid receptor [4,5] because of its high abundance in the central nervous system to modulate neurotransmission [6]. CB_1_ has also been identified in peripheral organs and tissues such as adipose tissue, skeletal muscles, gut, and pancreatic islets [7,8,9,10,11]. The CB_2_ is mainly located in immune cells such as macrophages, NK-cells, and B-lymphocytes, but is also expressed in brainstem [12] and other brain regions [13,14]. In liver tissue, controversial results show expression of both receptor types, while others could only find very low or no expression [15,16]. While the distribution of these two receptors in various tissues has been well studied, the changes of their expression during light and dark phases are unknown. There is increasing evidence that pharmacological alteration of cannabinoid receptor (CBR) signaling affects processes regulated in a cyclical, circadian or diurnal manner, for instance sleep/wake cycles [17], food consumption or fat storage [18] and endocrine functions [19].

The diurnal metabolic rhythms display an important temporal dimension of metabolic homeostasis in mammals. In the liver, the daily fluctuations of metabolites are accompanied by the cyclic activation of major metabolic pathways, including gluconeogenesis, de novo lipogenesis, lipoprotein secretion and glycolysis (review [20]). For example, the glycogen metabolism during the diurnal cycle is mediated by changes in the activities of enzymes such as phosphorylase and glycogen synthase (review [21]). Furthermore, the hepatic glucose metabolism is under the control of insulin that by itself showed oscillations during the day in young and middle-aged rats [22]. A recent study revealed that the loss of diurnal rhythm of circulating insulin concentration by high fat diet was closely associated with disappearance of diurnal expression of lipogenic and clock genes in the liver of mice [23]. Whether this loss of diurnal rhythm is also associated with different CBRs expression during a light/dark cycle in the liver has not yet been investigated.

Recent studies indicate the particular importance of CBRs in mediating a number of biological functions in different types of liver cells [24] and the modulation of insulin sensitivity by endocannabinoids [25]. Increased hepatic glucose concentration may result from a CB_1_-mediated increase in glycogenolysis [26] and/or gluconeogenesis [27]. In this context, the over-activity of CB_1_ receptor contributes to the development of insulin resistance of both, type 1 and 2 diabetes [28,29]. In addition, the CB_2_ receptor seems to counteract the inflammatory response in fatty tissue and in the liver. Thus, CB_2_ deficient mice exhibit obesity with hypertrophy of visceral fat and immune cell polarization towards pro-inflammatory subpopulations in the liver [30]. On the other hand, CB_1_ receptor blockade leads to a marked reduction in food intake and body fat mass, concomitant with improved insulin sensitivity and glucose tolerance. This effect was present in older mice, but not in younger animals, indicating age-dependent effects of CB_1_ receptor inhibition upon metabolic function in vivo [31]. Insulin sensitivity and insulin-stimulated glucose uptake in skeletal muscle was enhanced in 12-month-old CB_2_^−/−^ mice compared to 2-month-old mice [32]. Interestingly, another study showed an impact of aging on insulin oscillations during the day, in which only middle-aged rats exhibited a distinct diurnal rhythm [22].

Considering the liver as a central organ which is involved in the regulation of glucose metabolism and is a potential target of the metabolic actions of the endocannabinoid system [33,34], the issue of diurnal changes especially in younger and older individuals is of high relevance taking the raising rate of diabetes in older individuals into account. Therefore, the present study will focus on analysis of daytime-dependent expression of cannabinoid receptors *Cnr1* and *Cnr2* in the liver of rats to investigate the impact of diurnal rhythm on receptor expression. The use of younger and older rats will allow for determining the influence of aging on daytime-dependent expression patterns of CBRs based on differences in plasma insulin concentrations during the day as previously shown [22]. Furthermore, the influence of insulin on the daily expression of hepatic CBRs is analyzed in streptozotocin (STZ)-induced type 1 diabetes rats.

## 2. Results

RT-PCR was performed to analyze the tissue specific distribution of cannabinoid receptors 1 and 2 using specific primers (*Cnr1* and *Cnr2*, Table 1) as indicated by restriction analysis.

**Figure 1:**
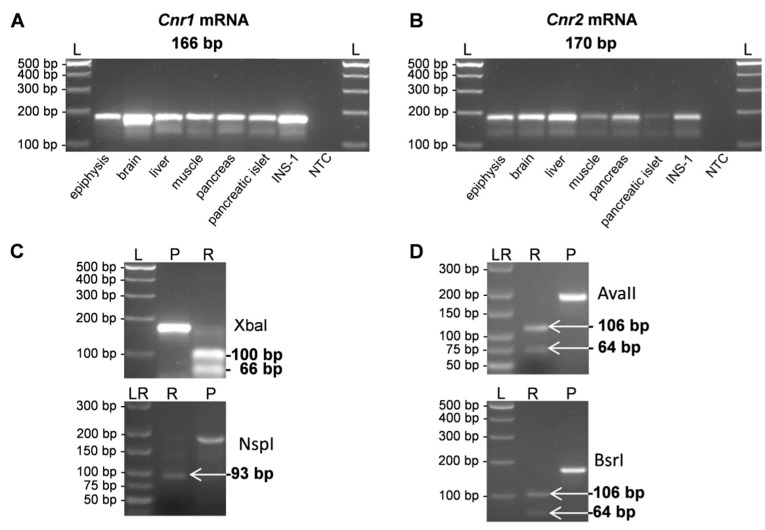
(**A**,**B**) RT-PCR products of cannabinoid receptors after gel-electrophoretic separation on 3% agarose gels, providing evidence that the transcripts were present in different organs. Molecular sizes of the respective PCR products *Cnr1* (**A**) and *Cnr2* (**B**) are indicated relative to a molecular size standard (L); (**C**) Restriction digestion of the receptor transcript *Cnr1* (P) using the restriction enzyme Xbal shows the predicted restriction fragments of 100 bp and 66 bp (R). After application of the restriction enzyme NspI a fragment with the predicted length of 93 bp becomes visible (R). Please note that smaller fragments (14 bp, 22 bp, and 37 bp) are not seen; (**D**) Restriction fragments of the *Cnr2* receptor product (P) after incubation with the restriction enzymes AvaII or BsrI predicted lengths of 64 bp und 106 bp (R) are detected, respectively. NTC, non-template control; L, 100-bp ladder; LR, low range ladder.

The transcripts of *Cnr1* and *Cnr2* were detected in epiphysis, brain, liver, muscle, pancreas, pancreatic islet and INS-1 cells with specific amplification product size of rat *Cnr1* at 166 bp and rat *Cnr2* at 170 bp (Figure 1A,B). Pancreatic islets and liver are co-players in the glucose metabolism. Therefore, INS-1 cells are used as an appropriate and generally accepted β-cell model for in vitro analyses and transcript detections [35].

The identity of the amplification product of each receptor was successfully confirmed by restriction analysis (Figure 1C,D). Restriction reaction using the restriction enzymes XbaI or NspI and AvaII or BsrI resulted in defined fragments of *Cnr1* amplification products with 100 bp and 66 bp or 93 bp (Figure 1C) molecular sizes and of *Cnr2* amplification products with 64 bp and 106 bp (Figure 1D).

**Figure 2:**
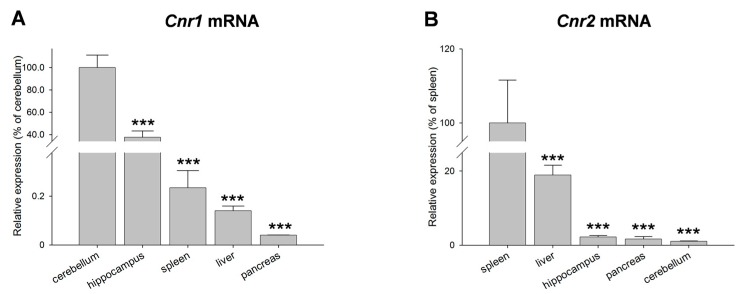
(**A**,**B**) Relative expression levels of *Cnr1* and *Cnr2* transcripts in cerebellum, hippocampus, spleen, liver and pancreas as means (±S.E.M.) of *n* = 4–6 rats, *** *p* < 0.001.

Semi-quantitative analysis of *Cnr1* and *Cnr2* in different rat tissues showed very high expression levels of *Cnr1* in cerebellum and hippocampus. The relative expression in cerebellum was set to 100% and compared to *Cnr1* expression in other organs. When comparing the peripheral organs a relative strong expression was evident in the liver (Figure 2A). *Cnr2* was highly expressed in spleen. The relative expression of *Cnr2* was set to 100%. In comparison to other investigated organs, the liver showed a relatively strong expression. *Cnr2* levels were low in cerebellum, hippocampus and pancreas (Figure 2B).

Therefore, both receptors were present in liver tissue and the issue whether diurnal changes are present was raised. To analyze the influence of daytime changes on the expression of liver cannabinoid receptor mRNA and protein samples were investigated every 3 h during a 24-h period.

**Figure 3:**
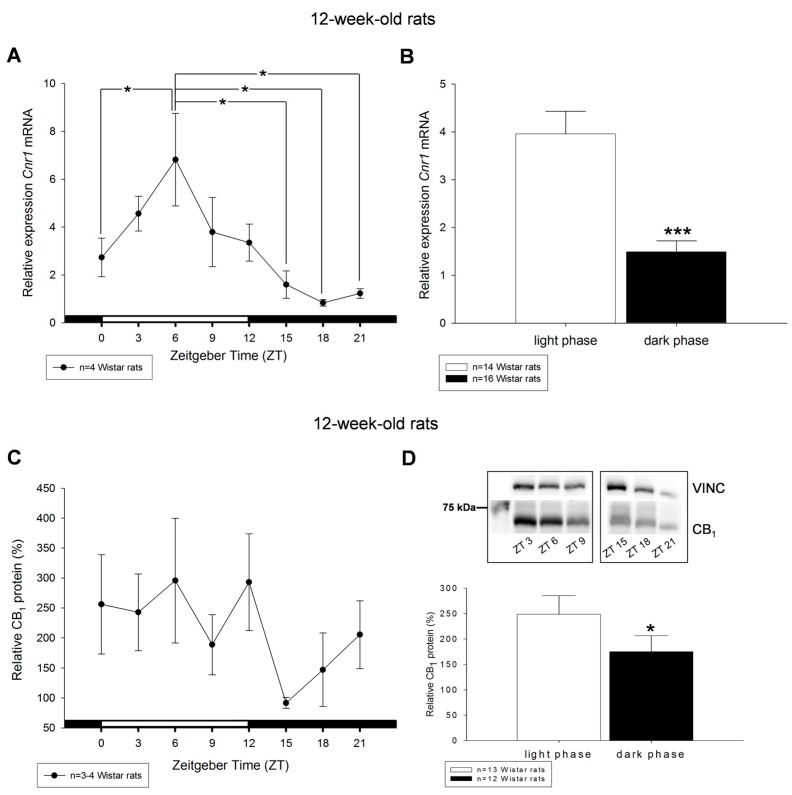
Diurnal profiles of cannabinoid receptor *Cnr1* mRNA (**A**) and CB_1_ protein (**C**) in liver of 12-week-old rats are shown. Diurnal differences were analyzed by Mann–Whitney test; * *p* < 0.05, *** *p* < 0.001. Values of light phase as well as those of dark phase are summarized for *Cnr1* mRNA (**B**) and CB_1_ protein (**D**). The insets in (**D**) illustrate the detection of single band of 64 kDa of CB_1_ via Western blotting in liver tissue obtained from the middle of the day respective night. Vinculin (VINC) was used as control protein.

Livers from 12-week-old rats exhibited a diurnal rhythm of *Cnr1* mRNA levels with highest values at ZT3, ZT6 and ZT9 and lowest during the dark period at ZT18 and ZT21 (Figure 3A). Statistically significant differences were found with higher levels of mRNA during the light period compared to transcript levels during dark period (Figure 3B). Similar results were found when protein expression of the light and dark phase was compared. We detected higher temporal variations of hepatic CB_1_ protein levels per daytime point in 12-week-old animals (Figure 3C,D). In summary, CB_1_ protein levels for light and dark phase showed statistically significant difference between the day and night periods (Figure 3D and Figure S1A), with lower CB_1_ protein levels during dark phase.

**Figure 4:**
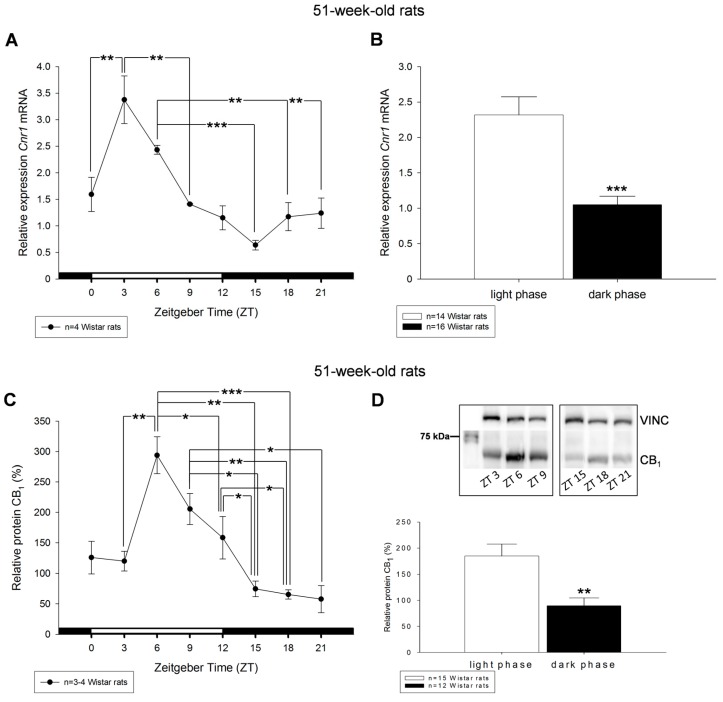
Diurnal profiles of cannabinoid receptor *Cnr1* mRNA (**A**) and CB_1_ protein (**C**) in liver of 51-week-old rats are shown. Diurnal differences were analyzed by Mann–Whitney test; * *p* < 0.05, ** *p* < 0.01, *** *p* < 0.001. Values of light phase as well as those of dark phase are summarized for *Cnr1* mRNA (**B**) and CB_1_ protein (**D**). The insets in (**D**) illustrate the detection of single band of 64 kDa of CB_1_ via Western blotting in liver tissue obtained from the middle of the day respective night. Vinculin (VINC) was used as control protein.

The 51-week-old rats exhibited a more distinct, statistically significant diurnal rhythm for *Cnr1* as well as CB_1_ protein, with highest levels during the early light phase (Figure 4A,C). Highest peak for *Cnr1* was measured at ZT3, for CB_1_ protein at ZT6. In general, transcript expression and protein content of CB_1_ were significantly increased during the light period and diminished during darkness (Figure 4B,D and Figure S1B).

**Figure 5:**
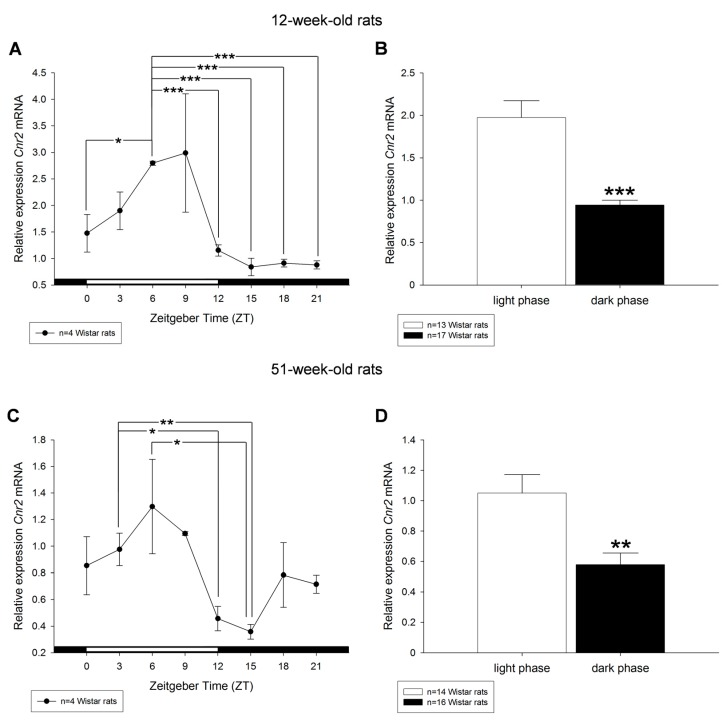
Diurnal profiles of cannabinoid receptor *Cnr2* mRNA expression in liver of 12-week-old (**A**) and 51-week-old (**C**) rats are shown. Diurnal differences were analyzed by Mann–Whitney test; * *p* < 0.05, ** *p* < 0.01, *** *p* < 0.001. Values of light phase as well as those of dark phase are summarized for *Cnr2* mRNA in 12-week-old (**B**) and 51-week-old rats (**D**).

The diurnal expression patterns of *Cnr2* transcript in the liver of 12- and 51-week-old rats revealed for both groups highest values at ZT6 and ZT9 during light period. Lower *Cnr2* transcript levels were measured during the dark period displaying lowest peak at ZT15 (Figure 5A,C). In summary, transcript levels of *Cnr2* were decreased during the dark phase compared to the light phase regardless of the age (Figure 5B,D). Both hepatic cannabinoid receptor type 1 and type 2 showed a diurnally regulated expression pattern in 12- and 51-week-old Wistar rats.

**Figure 6:**
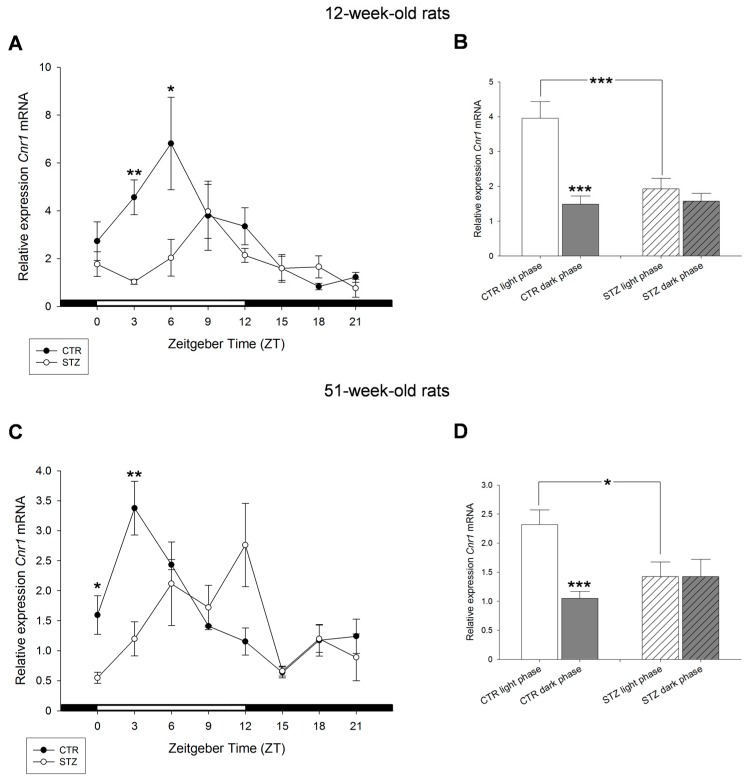
Effects of streptozotocin on the diurnal expression of cannabinoid receptor *Cnr1* mRNA in the liver of: 12-week-old rats (**A**,**B**); and 51-week-old rats (**C**,**D**). (**A**,**C**) Streptozotocin treated Wistar rats (STZ) are shown compared to control Wistar rats (CTR). (**B**,**D**) Values of light phase as well as those of dark phase are summarized for *Cnr1* mRNA of control Wistar rats (CTR) and streptozotocin treated rats (STZ) showing decreased expression of *Cnr1* mRNA in STZ rats during light period compared to control. Values are means (±S.E.M.), as analyzed by Mann–Whitney test. * *p* < 0.05, ** *p* < 0.01, *** *p* < 0.001.

A model of type 1 diabetes was induced by streptozotocin (STZ) treatment, which is characterized by a nearly total loss of insulin producing beta cells, insulin secretion and significantly increased blood glucose concentrations ([22], Figure S2). In 12- and 51-week-old STZ rats, the *Cnr1* mRNA receptor expression was significantly diminished at two time points during light period compared to controls: in 12-week-old animals at ZT3 and in 51-week-old rats at ZT0 and ZT3 (Figure 6A,C). There was a significant decrease of *Cnr1* mRNA under diabetic state compared to control rats during light phase in 12- and 51-week-old rats (Figure 6B,D). In livers of 12-week-old rats this decrease was more pronounced when compared to 51-week-old rats since in older rats an increase at ZT12 was seen. In addition, the significant difference between light and dark period measured in both, 12- and 51-week-old rats (Figure 3 and Figure 4), could not be observed in STZ rats (Figure 6B,D).

**Figure 7:**
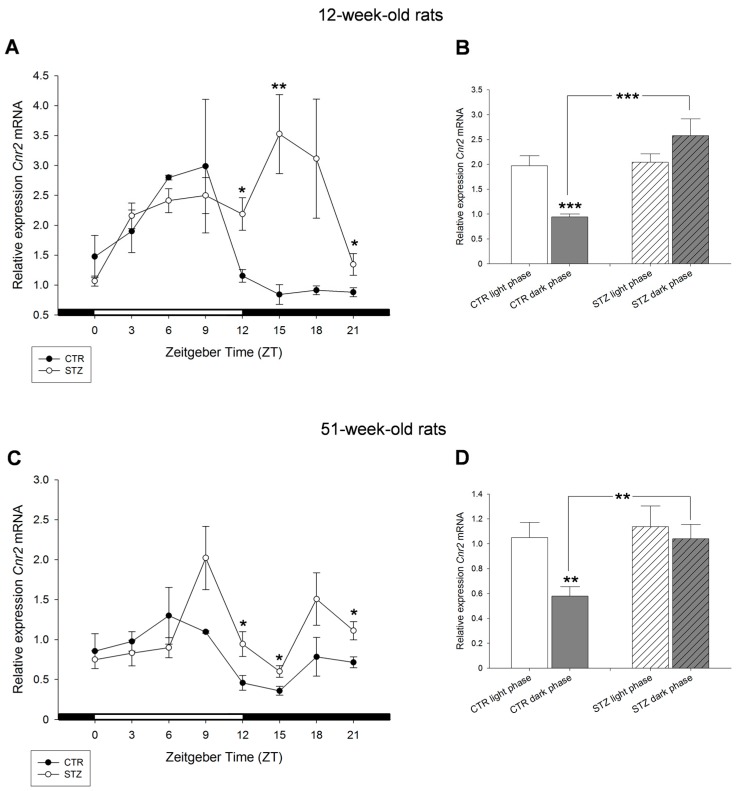
Effects of streptozotocin on the diurnal expression of cannabinoid receptor *Cnr2* mRNA in the liver of: 12-week-old rats (**A**,**B**); and 51-week-old rats (**C**,**D**). (**A**,**C**) Streptozotocin treated Wistar rats (STZ) compared to control Wistar rats (CTR). (**B**,**D**) Values of light phase as well as those of dark phase are summarized for *Cnr2* mRNA of control Wistar rats (CTR) and streptozotocin treated rats (STZ) showing increased expression of *Cnr2* mRNA in STZ rats during dark period compared to control. Values are means (±S.E.M.), as analyzed by Mann–Whitney test. * *p* < 0.05, ** *p* < 0.01, *** *p* < 0.001.

The comparison of diurnal profiles of *Cnr2* mRNA of 12- and 51-week-old control and STZ rats showed significant differences during the dark phase. Significantly increased levels of *Cnr2* mRNA were detected in STZ animals of both ages at ZT12 and ZT15 when compared to controls (Figure 7A,C). *Cnr2* mRNA was significantly increased during dark phase in diabetic rats compared to control. Interestingly, the day–night variations of *Cnr2* observed in control animals were not present in STZ rats (Figure 7B,D).

Taking together, in STZ rats the absence of insulin that is associated with increased blood glucose concentration (Figure S2) leads to a loss of daily fluctuations of *Cnr1* as well *Cnr2* in both 12- and 51-week-old animals.

## 3. Discussion

The liver plays a central role in the control of energy metabolism and bodyweight regulation [36]. There is growing evidence that physiological control of food intake and energy metabolism is in part under control of the endocannabinoid system (ECS), through its ability to target central and peripheral sites including the liver [37]. It is well known that cannabinoid receptors (CBRs) as well as other components of ECS are present in liver tissue [25]. While expression of CB_1_ receptors has been shown in almost all hepatic cells, such as hepatocytes, stellate and endothelial cells; hepatocytes are in addition able to produce endocannabinoids by itself [8,25,38,39,40,41]. CB_2_ receptors are expressed in stellate cells and Kupffer cells [42]. In accordance, CB_1_ and CB_2_ were detected in the liver tissue in the present study, which is in contrast to some studies describing only low or absent CBR expression in the liver [15,16] and hepatocytes [8]. In our study, *Cnr* transcripts were detectable in substantial amounts in liver tissue when compared to other organs. Highest amount of *Cnr1* was observed in cerebellum and hippocampus, although expression in cerebellum was higher compared to hippocampus. This finding is in accordance with a previous study reporting about higher *Cnr1* expression levels in cerebellum than hippocampus in rats [43]. Nonetheless, it should be noted that, at protein level, controversial data exist. Whereas some authors found similar levels of CB_1_ in cerebellum and hippocampus [4], others described highest CB_1_ density in cerebellar cortex and cerebellum [44]. Considering the daytime dependent expression of CBR mRNA and protein, the current data show that in the liver the cannabinoid receptor transcripts *Cnr1* and *Cnr2* underlie daily variations. The diurnal rhythm of cannabinoid receptors is mainly the result of: (a) food intake, which is associated with rhythmic expression of the vast majority of hepatic genes [45]; and (b) circadian regulation since approximately 20% of liver-soluble proteins underlie the control of circadian master clock [46]. As mentioned above, food intake by itself can function as a potent Zeitgeber for peripheral tissues as such the liver, underscoring the important relationship between circadian and metabolic processes [47].

Our findings provide strong evidence that the expression of CB_1_ and CB_2_ gene products depends on diurnal variations displaying higher levels during light period and decreased expression during darkness. These diurnal receptor level variations are also reported in rat brain showing substantial diurnal variation of CB_1_ mRNA expression and protein content over the 24-h period in the pons of rats, which were under a controlled light/dark cycle 12:12 [48]. These data indicated a maximum protein expression of CB_1_ at ZT5, a maximum mRNA expression at ZT13, and minimum expression values at ZT17 and ZT1, respectively. In addition, CB_1_ expression varied in the hippocampus, being higher at ZT5 hours and lower at ZT17, whereas its expression remained unchanged in the striatum [49].

An interesting relationship was hypothesized between changes in endocannabinoid content and CB_1_ density with time of the day in pons and hippocampus. CB_1_ density was high during light period paralleled by low AEA and high 2-AG contents allowing high efficacy of 2-AG to activate CB_1_ signaling [50]. Such differences in the efficacy of CBR agonists or antagonists might be explained by the differential diurnal expression pattern of their receptors.

Considering the diurnal expression of CBRs in the liver, it is well recognized that endocannabinoids including their CBRs contribute to the metabolic regulation of glucose homeostasis, lipid metabolism and other metabolic functions in the liver [9,28].

The temporal pattern of food intake and the circadian clock drive rhythmic transcription in hepatic genes [45]. The consumption of a high-caloric diet in mice alters the function of the mammalian circadian clock in the hypothalamus, liver and adipose tissue [51]. In this regard, it is well established, that the hepatic endocannabinoid system and especially the CB_1_ receptor are activated in response to high fat feeding (diet induced obesity). Activation of hepatocyte CB_1_ inhibits the insulin clearance by insulin degrading enzymes, thus contributing to hyperinsulinemia and increased glycogenosis [26]. In addition, CB_1_ promotes lipogenesis in mouse hepatocytes and in human liver cells [24]. Activation of both cannabinoid receptors, CB_1_ and CB_2_, promote the development of obesity, insulin resistance and dyslipidemia [52,53,54]. According to Maccarrone et al. [24], CB_1_ and CB_2_ seemingly act in opposite directions in the liver: CB_1_ promotes fibrogenesis and steatosis, whereas CB_2_ is generally believed to mediate beneficial effects of endocannabinoids and prevents liver dysfunction. Despite of the well-known contributions of both receptors to liver diseases such as steatosis the physiological relevance of these receptors in the liver is less clear and needs further investigation. CB_1_ receptor signaling has been shown to regulate the hepatic gluconeogenesis. 2-AG treatment in primary rat hepatocytes significantly induced mRNA levels of the key gluconeogenic enzyme genes Pepck (phosphoenolpyruvate carboxykinase) and G6pc (glucose-6-phosphatase catalytic subunit) in a time-dependent manner and caused significant increase in glucose production in hepatocyte culture media [27]. Interestingly, hepatic glucose regulatory genes also displayed a time-of-day dependent expression pattern in the mouse liver [55]. Genes such as glucokinase, pyruvate kinase, phosphoenolpyruvate kinase 1 and glucose-6-phosphate translocase were upregulated during the light-phase [55], which is paralleled by hepatic CBR upregulation in our study. Similarly, a diurnal variation of lipogenic enzyme mRNA levels in rat livers were reported [56]. Key lipogenic enzymes fatty acid synthetase, acetyl-CoA-carboxylase and ATP-citrate lyase showed diurnal variations reaching their maximum activity at night and minimum during the light period [57]. Indeed, the CB_1_ agonist HU210 induced lipogenic gene expression and de novo hepatic fatty acid synthesis in mouse liver in vivo or in vitro [8,24]. Taken together, these findings strongly imply that, the diverse patterns of hepatic CBR changes during the day seem to be associated with changes of hepatic glucose and lipid metabolism. Previous reports focused on the age-related deregulation of CBRs and endocannabinoid hydrolyzing enzymes in rat cerebral cortex as another aspect of receptor modulation [58,59]. For the liver it has been shown that CB_1_-antagonist rimonabant improved insulin sensitivity and glucose tolerance in aged, but not young adult mice. This indicates that aging seems to be a relevant factor on efficacy of the endocannabinoid system on glucose metabolism [31]. In our study, the protein profile displayed more clearly a diurnal variation pattern in 51-week-old than in 12-week-old rats. Accordingly, plasma insulin levels of both 12- and 51-week-old rats showed oscillations during the day. However, the 51-week-old rats exhibited a distinct, statistically significant diurnal rhythm, with higher levels in the late afternoon and during the first half of the dark period [22]. A diurnal rhythm was found for hepatic InsR transcripts in 12-week-old rats, whereas, in 51-week-old rats, the amplitude was diminished and the phase was shifted (unpublished results). The observed discrepancy on *Cnr1*/*Cnr2* expression in 12- vs. 51-week-old rats could also be explained by differences in the peripheral endocannabinoid levels. Increased systemic 2-AG concentrations in visceral obese humans were associated with decreased *Cnr1* receptor gene expression in adipose tissue, suggesting a negative-feedback loop [60]. The direct comparison of *Cnr1* receptor expression between 12- and 51-week-old rats revealed decreased *Cnr1* expression in older rats (unpublished results) with higher body weights (Figure S2A). These findings suggest that expression of CBR during the daytime is influenced by the aging process, which should be included in further considerations to explain receptor functions.

To further explore the relevance of the diurnal and age-related changes in expression of CBR we studied the streptozotocin (STZ) model in 12- and 51-week-old rats. STZ is a widely used animal model to induce experimental type I diabetes. In livers obtained from STZ-rats the typical diurnal expression pattern of both CB_1_ and CB_2_ receptors were lost when compared to control rats of both ages. This indicates an insulin dependent regulation of CBR expression. In accordance with our results, in certain brain regions of STZ rats, CBR expression and phosphorylation status were altered, although diurnal receptor expression pattern was not investigated in this study [61]. In contrast to our study with lower *Cnr1* expression, increased *Cnr1* levels have previously been reported in hepatic and pancreatic tissues in STZ treated rats [62]. This discrepancy might be explained by different experimental conditions. While in the experiments of You et al. [62] STZ (60 mg per kg body weight) STZ was injected for one week, we used as single intraperitoneal application of the same STZ dose and investigated the liver 6 weeks later. Furthermore, the plasma insulin level was significantly decreased in the former study [62], whereas in our rats almost a loss of insulin plasma was measured ([22], Figure S2B).

Different from CB_1_, the CB_2_ receptor expression was increased in liver of diabetic STZ rats compared to control animals during the dark phase. CB_2_ receptors have been shown to increase inflammation with anti-fibrogenic properties [63]. In addition to CB_1_ and CB_2_ effects in the liver, numerous studies have demonstrated beneficial effects of cannabidiol, a potent phytocannabinoid, in primary diabetes and different diabetic complications [64]. Treatment with cannabidiol significantly prolongs the disease outbreak (from 86% in non-treated control mice to 30% in cannabidiol-treated mice) of non-obese-diabetes (NOD) mice [65]. Thus, a preventive role of cannabidiol in development of human type 1 diabetes is suggested [66], although cannabidiol itself has no affinity for CB_1_, however, several of its hydrogenated analogs bind with nanomolar affinity [67]. Pharmacological manipulations of the CBRs were postulated to improve and to normalize glucose homeostasis in diabetes associated with obesity [52]. The diurnal expression profiles of hepatic cannabinoid receptors under diabetic conditions and in aging should be considered when modulation of the endocannabinoid system is planned in therapeutic conditions. The diurnal influence might help especially in older age to achieve a more effective impact of endocannabinoids on hepatic metabolism.

In conclusion, we have shown diurnal changes of cannabinoid receptor type 1 and type 2, which are of significant physiological and clinical interest. Considering the diurnal rhythm, the variation of CBR expression seems to be predominantly driven by food entrainment. Diurnal receptor changes are influenced by age. They are lost in disease model of type I diabetes.

## 4. Materials and Methods

### 4.1. Animals and Tissue Collection

Experiments were performed with male Wistar rats (Outbred; Schönwalde, Germany), which were kept in type III Macrolon^®^ cages in groups of three animals, with controlled temperature (22 ± 1 °C) and humidity (50–60%), and subjected to a light regime of light:dark, L:D = 12:12 (lights on at 07:00 a.m. defined as ZT0 = being the beginning of the rest period; ZT12 being the beginning of the activity period, mid-darkness defined as ZT18; light off at 07:00 p.m. defined as ZT12).

Rats were fed a standard diet (Altromin 1314; Altromin, Lage, Germany), whereby food and water were available ad libitum. During all experimental procedures, the animals were treated according to European welfare regulations.

Control group of normoglycemic male Wistar rats, 40 young rats (12 weeks) and 40 middle-aged rats (51 weeks) were examined (Figure S2). In parallel (STZ group), male Wistar rats at an age of six weeks or at an age of 45 weeks were treated with a single dose of 60 mg STZ/kg body weight (Calbiochem, San Diego, CA, USA) to induce type I diabetes [22]. Animals were treated according to German animal welfare regulations throughout the sampling procedure (permission of the Landesverwaltungsamt, Sachsen-Anhalt, Germany: 2-737 MLU). The young control or STZ rats were sacrificed by heart ventricle puncture at the age of 12 weeks, and the middle-aged control or STZ rats at the age of 51 weeks every 3 h (*n* = 5) beginning at 7:00 a.m. = ZT0. Tissue sampling of liver proceeded under red light during the dark phase. In addition, from young control Wistar rats other organs (hippocampus, cerebellum, spleen and pancreas) were obtained for RNA extraction and subsequent semi-quantification analysis.

### 4.2. RNA Extraction, DNase 1 Digestion and Reverse Transcription

To check the presence of transcripts RNA was extracted from frozen tissue of liver, other organs (skeletal muscle, brain, spleen, pancreas, epiphysis, pancreatic islets) and INS-1 cells (Ambion Inc., Austin, TX, USA) using a Trizol-based extraction method according to the manufacturer’s instructions (Invitrogen Inc., Carlsbad, CA, USA). Total RNA concentrations were determined by spectrophotometry at 260 nm and RNA quality assessed by electrophoresis on 1.3% denaturizing formaldehyde agarose gels. For the elimination of residual DNA, total RNA was subsequently subjected to DNase I digestion (DNA-free™, Ambion Inc.). Total RNA of 1 µg was reverse transcribed using reverse-transcription kit from Promega (Promega Inc., Madison, WI, USA) according to the manufacturer’s protocol.

### 4.3. Semi-Quantitative, Real-Time RT-PCR

Real-time reverse transcription-polymerase chain reaction (RT-PCR) was performed using reaction volumes of 20 µL containing 40 ng cDNA, 10 µL master mix (Promega Inc., Madison, WI, USA), 0.5 µL of each primer (25 pM), 0.25 µL of a fluorescence dye (Eva Green, Biotrend Chemicals, UC, Destin, FL, USA) and 4,75 µL RNA-free water. Real-time RT-PCR was carried out using a rotor-cycler (Rotor-GeneTM RG 6000, Corbett Research, Pty Ltd., Sydney, Australia). The PCR conditions were: initial denaturation at 95 °C, followed by 40 cycles of denaturation at 94 °C, annealing at 64 °C (followed by a stepwise decrease in 1 °C per cycle during the first five cycles down to an annealing temperature of 59 °C), elongation at 72 °C and fluorescence detection at 80 °C. Relative mRNA concentrations of transcripts were calculated according to the quantification method using β-actin as a reference gene for normalization of transcript expression [68,69]. In addition, the β-actin was shown by others to remain unaltered during circadian period [70,71]).

Sequences of primers used in the present study and their amplicon sizes are listed in Table 1. Restriction enzymes (XbaI, NspI, AvaII, and BsrI) to determine specificity of amplification products are from Promega GmbH (Mannheim, Germany) and Fermentas GmbH (St. Leon-Rot, Germany). The rodent nomenclature of genes (*Cnr1* and *Cnr2*) was used for CB_1_ and CB_2_.

### 4.4. Western Blot

Protein extracts of liver tissue were obtained by cell lysis buffer. The total protein was measured by Pierce BCA Protein Assay Kit (ThermoScientific, Rockford, IL, USA). Samples containing 50 µg/µL total protein of tissue lysates were loaded with 5× sample buffer separated by a 10% SDS-polyacrylamide gel electrophoresis and transferred onto nitrocellulose membranes (Millipore Immobilon^®^-P, Carl Roth GmbH, Karlsruhe, Germany) using standard protocols.

Membranes were blocked for 1 h with 1% Roti-block solution (Carl Roth GmbH, Karlsruhe, Germany) or 5% milk in wash buffer and incubated overnight at 4 °C with following primary antibodies: guinea pig polyclonal anti CB_1_ antibody (1:1000, CB1-Go-Af530, Frontier Institute, Hokkaido, Japan) or loading control rabbit monoclonal anti-vinculin antibody (1:5000; ab129002, Abcam, Caimbridge, UK). Molecular weight of mature CB_1_ receptors was determined as reported [72]. Membranes were subsequently washed and the horseradish peroxidase-conjugated secondary antibodies anti-guinea pig immunoglobulins (P0141, 1:1000; DAKO, Hamburg, Germany) or anti-rabbit IgG (1:1000; Cell Signaling Technology, Danvers, MA, USA) were applied for 1 h at room temperature.

Chemiluminescence detection system Western Bright Quantum (K-12042-D20, Advansta Inc., Menlo Park, CA, USA) was used to visualize immunoreactive bands. Fusion FX7 software (Peqlab, Erlangen, Germany) was used to digitize immunoreactive bands and quantification of Western-Blot signals was done with Fusion Bio1D software (Peqlab, Erlangen, Germany). Intensities of individual protein bands were normalized against the vinculin signal. The specificity of CB_1_ antibody was given as reported earlier [73,74].

### 4.5. Statistics

For statistical evaluation of results and significance testing of group differences, a Mann–Whitney test was performed using GraphPad Prism software (Version 5.0, GraphPad Software Inc., San Diego, CA, USA) and graphs were generated using Sigma Plot 8.0 software (SPSS Inc., Chicago, IL, USA) and GraphPad Prism software. Data were presented as means (and ±S.E.M.). Groups were considered to be significantly different at *p* < 0.05 for a parameter.

## Figures and Tables

**Table 1:** Rat primer and sequences.

Gene	Forward Primer (5’–3’)	Reverse Primer (5’–3’)	Amplicon Size (bp)	GenBank ID
*Cnr1*	AGGAGCAAGGACCTGAGACA	TAACGGTGCTCTTGATGCAG	166	NM_012784.4
*Cnr2*	AGGTTGCATTCCCAACAGAC	TTAGTTCCTCTGGGCAATGG	170	NM_001164143.2
*β-actin*	ACTCCTACGTGGGCGACGAGG	CAGGTCCAGACGCAGGATGGC	389	NM_031144

## Supplementary Materials

Supplementary materials can be found at www.mdpi.com/1422-0067/18/9/1844/s1.

## References

[1] Di Marzo V. (2008). CB_1_ receptor antagonism: Biological basis for metabolic effects. Drug Discov. Today.

[2] Matsuda L.A., Lolait S.J., Brownstein M.J., Young A.C., Bonner T.I. (1990). Structure of a cannabinoid receptor and functional expression of the cloned cDNA. Nature.

[3] Munro S., Thomas K.L., Abu-Shaar M. (1993). Molecular characterization of a peripheral receptor for cannabinoids. Nature.

[4] Herkenham M., Lynn A.B., Johnson M.R., Melvin L.S., de Costa B.R., Rice K.C. (1991). Characterization and localization of cannabinoid receptors in rat brain: A quantitative in vitro autoradiographic study. J. Neurosci..

[5] Devane W.A., Hanus L., Breuer A., Pertwee R.G., Stevenson L.A., Griffin G., Gibson D., Mandelbaum A., Etinger A., Mechoulam R. (1992). Isolation and structure of a brain constituent that binds to the cannabinoid receptor. Science.

[6] Dalton G.D., Bass C.E., van Horn C.G., Howlett A.C. (2009). Signal transduction via cannabinoid receptors. CNS Neurol. Disord. Drug Targets.

[7] Massa F., Storr M., Lutz B. (2005). The endocannabinoid system in the physiology and pathophysiology of the gastrointestinal tract. J. Mol. Med..

[8] Osei-Hyiaman D., DePetrillo M., Pacher P., Liu J., Radaeva S., Bátkai S., Harvey-White J., Mackie K., Offertáler L., Wang L. (2005). Endocannabinoid activation at hepatic CB_1_ receptors stimulates fatty acid synthesis and contributes to diet-induced obesity. J. Clin. Investig..

[9] Cota D. (2007). CB_1_ receptors: Emerging evidence for central and peripheral mechanisms that regulate energy balance, metabolism, and cardiovascular health. Diabetes Metab. Res. Rev..

[10] Tharp W.G., Lee Y.-H., Maple R.L., Pratley R.E. (2008). The cannabinoid CB_1_ receptor is expressed in pancreatic delta-cells. Biochem. Biophys. Res. Commun..

[11] O’Keefe L., Simcocks A.C., Hryciw D.H., Mathai M.L., McAinch A.J. (2014). The cannabinoid receptor 1 and its role in influencing peripheral metabolism. Diabetes Obes. Metab..

[12] Van Sickle M.D., Duncan M., Kingsley P.J., Mouihate A., Urbani P., Mackie K., Stella N., Makriyannis A., Piomelli D., Davison J.S. (2005). Identification and functional characterization of brainstem cannabinoid CB_2_ receptors. Science.

[13] Skaper S.D., Buriani A., Dal Toso R., Petrelli L., Romanello S., Facci L., Leon A. (1996). The ALIAmide palmitoylethanolamide and cannabinoids, but not anandamide, are protective in a delayed postglutamate paradigm of excitotoxic death in cerebellar granule neurons. Proc. Natl. Acad. Sci. USA.

[14] Núñez E., Benito C., Pazos M.R., Barbachano A., Fajardo O., González S., Tolón R.M., Romero J. (2004). Cannabinoid CB_2_ receptors are expressed by perivascular microglial cells in the human brain: An immunohistochemical study. Synapse.

[15] Buckley N.E., Hansson S., Harta G., Mezey E. (1998). Expression of the CB_1_ and CB_2_ receptor messenger RNAs during embryonic development in the rat. Neuroscience.

[16] Teixeira-Clerc F., Julien B., Grenard P., van Tran Nhieu J., Deveaux V., Li L., Serriere-Lanneau V., Ledent C., Mallat A., Lotersztajn S. (2006). CB_1_ cannabinoid receptor antagonism: A new strategy for the treatment of liver fibrosis. Nat. Med..

[17] Murillo-Rodríguez E. (2008). The role of the CB_1_ receptor in the regulation of sleep. Prog. Neuro-Psychopharmacol. Biol. Psychiatry.

[18] De Kloet A.D., Woods S.C. (2009). Minireview: Endocannabinoids and their receptors as targets for obesity therapy. Endocrinology.

[19] Maccarrone M., Wenger T. (2005). Effects of cannabinoids on hypothalamic and reproductive function. Handb. Exp. Pharmacol..

[20] Li S., Lin J.D. (2015). Transcriptional control of circadian metabolic rhythms in the liver. Diabetes Obes. Metab..

[21] Agius L. (2015). Role of glycogen phosphorylase in liver glycogen metabolism. Mol. Asp. Med..

[22] Peschke E., Wolgast S., Bazwinsky I., Pönicke K., Muhlbauer E. (2008). Increased melatonin synthesis in pineal glands of rats in streptozotocin induced type 1 diabetes. J. Pineal Res..

[23] Honma K., Hikosaka M., Mochizuki K., Goda T. (2016). Loss of circadian rhythm of circulating insulin concentration induced by high-fat diet intake is associated with disrupted rhythmic expression of circadian clock genes in the liver. Metabolism.

[24] Maccarrone M., Bab I., Bíró T., Cabral G.A., Dey S.K., Di Marzo V., Konje J.C., Kunos G., Mechoulam R., Pacher P. (2015). Endocannabinoid signaling at the periphery: 50 years after THC. Trends Pharmacol. Sci..

[25] Kunos G., Tam J. (2011). The case for peripheral CB_1_ receptor blockade in the treatment of visceral obesity and its cardiometabolic complications. Br. J. Pharmacol..

[26] Liu J., Zhou L., Xiong K., Godlewski G., Mukhopadhyay B., Tam J., Yin S., Gao P., Shan X., Pickel J. (2012). Hepatic cannabinoid receptor-1 mediates diet-induced insulin resistance via inhibition of insulin signaling and clearance in mice. Gastroenterology.

[27] Chanda D., Kim D.-K., Li T., Kim Y.-H., Koo S.-H., Lee C.-H., Chiang J.Y.L., Choi H.-S. (2011). Cannabinoid receptor type 1 (CB_1_R) signaling regulates hepatic gluconeogenesis via induction of endoplasmic reticulum-bound transcription factor cAMP-responsive element-binding protein H (CREBH) in primary hepatocytes. J. Biol. Chem..

[28] Silvestri C., di Marzo V. (2013). The endocannabinoid system in energy homeostasis and the etiopathology of metabolic disorders. Cell Metab..

[29] Mazier W., Saucisse N., Gatta-Cherifi B., Cota D. (2015). The endocannabinoid system: Pivotal orchestrator of obesity and metabolic disease. Trends Endocrinol. Metab..

[30] Schmitz K., Mangels N., Häussler A., Ferreirós N., Fleming I., Tegeder I. (2016). Pro-inflammatory obesity in aged cannabinoid-2 receptor-deficient mice. Int. J. Obes..

[31] Lipina C., Vaanholt L.M., Davidova A., Mitchell S.E., Storey-Gordon E., Hambly C., Irving A.J., Speakman J.R., Hundal H.S. (2016). CB_1_ receptor blockade counters age-induced insulin resistance and metabolic dysfunction. Aging Cell.

[32] Agudo J., Martin M., Roca C., Molas M., Bura A.S., Zimmer A., Bosch F., Maldonado R. (2010). Deficiency of CB_2_ cannabinoid receptor in mice improves insulin sensitivity but increases food intake and obesity with age. Diabetologia.

[33] Kunos G., Osei-Hyiaman D., Liu J., Godlewski G., Bátkai S. (2008). Endocannabinoids and the control of energy homeostasis. J. Biol. Chem..

[34] Nogueiras R., Diaz-Arteaga A., Lockie S.H., Velásquez D.A., Tschop J., López M., Cadwell C.C., Diéguez C., Tschöp M.H. (2009). The endocannabinoid system: Role in glucose and energy metabolism. Pharmacol. Res..

[35] Asfari M., Janjic D., Meda P., Li G., Halban P.A., Wollheim C.B. (1992). Establishment of 2-mercaptoethanol-dependent differentiated insulin-secreting cell lines. Endocrinology.

[36] Cooper M.E., Regnell S.E. (2014). The hepatic cannabinoid 1 receptor as a modulator of hepatic energy state and food intake. Br. J. Clin. Pharmacol..

[37] Pagotto U., Cervino C., Vicennati V., Marsicano G., Lutz B., Pasquali R. (2006). How many sites of action for endocannabinoids to control energy metabolism?. Int. J. Obes..

[38] Bátkai S., Járai Z., Wagner J.A., Goparaju S.K., Varga K., Liu J., Wang L., Mirshahi F., Khanolkar A.D., Makriyannis A. (2001). Endocannabinoids acting at vascular CB_1_ receptors mediate the vasodilated state in advanced liver cirrhosis. Nat. Med..

[39] Osei-Hyiaman D., Liu J., Zhou L., Godlewski G., Harvey-White J., Jeong W.-I., Bátkai S., Marsicano G., Lutz B., Buettner C. (2008). Hepatic CB_1_ receptor is required for development of diet-induced steatosis, dyslipidemia, and insulin and leptin resistance in mice. J. Clin. Investig..

[40] Siegmund S.V., Uchinami H., Osawa Y., Brenner D.A., Schwabe R.F. (2005). Anandamide induces necrosis in primary hepatic stellate cells. Hepatology.

[41] Siegmund S.V., Qian T., de Minicis S., Harvey-White J., Kunos G., Vinod K.Y., Hungund B., Schwabe R.F. (2007). The endocannabinoid 2-arachidonoyl glycerol induces death of hepatic stellate cells via mitochondrial reactive oxygen species. FASEB J..

[42] Siegmund S.V., Schwabe R.F. (2008). Endocannabinoids and liver disease. II. Endocannabinoids in the pathogenesis and treatment of liver fibrosis. Am. J. Physiol. Gastrointest. Liver Physiol..

[43] Berrendero F., García-Gil L., Hernández M.L., Romero J., Cebeira M., de Miguel R., Ramos J.A., Fernández-Ruiz J.J. (1998). Localization of mRNA expression and activation of signal transduction mechanisms for cannabinoid receptor in rat brain during fetal development. Development.

[44] Bidaut-Russell M., Devane W.A., Howlett A.C. (1990). Cannabinoid receptors and modulation of cyclic AMP accumulation in the rat brain. J. Neurochem..

[45] Vollmers C., Gill S., DiTacchio L., Pulivarthy S.R., Le H.D., Panda S. (2009). Time of feeding and the intrinsic circadian clock drive rhythms in hepatic gene expression. Proc. Natl. Acad. Sci. USA.

[46] Reddy A.B., Karp N.A., Maywood E.S., Sage E.A., Deery M., O’Neill J.S., Wong G.K.Y., Chesham J., Odell M., Lilley K.S. (2006). Circadian orchestration of the hepatic proteome. Curr. Biol..

[47] Eckel-Mahan K., Sassone-Corsi P. (2013). Metabolism and the circadian clock converge. Physiol. Rev..

[48] Martínez-Vargas M., Murillo-Rodríguez E., González-Rivera R., Landa A., Méndez-Díaz M., Prospro-García O., Navarro L. (2003). Sleep modulates cannabinoid receptor 1 expression in the pons of rats. Neuroscience.

[49] Rueda-Orozco P.E., Soria-Gomez E., Montes-Rodriguez C.J., Martínez-Vargas M., Galicia O., Navarro L., Prospero-García O. (2008). A potential function of endocannabinoids in the selection of a navigation strategy by rats. Psychopharmacology.

[50] Vaughn L.K., Denning G., Stuhr K.L., Wit H., de Hill M.N., Hillard C.J. (2010). Endocannabinoid signalling: Has it got rhythm?. Br. J. Pharmacol..

[51] Kohsaka A., Laposky A.D., Ramsey K.M., Estrada C., Joshu C., Kobayashi Y., Turek F.W., Bass J. (2007). High-fat diet disrupts behavioral and molecular circadian rhythms in mice. Cell Metab..

[52] Bermúdez-Silva F.J., Suárez Pérez J., Nadal A., Rodríguez de Fonseca F. (2009). The role of the pancreatic endocannabinoid system in glucose metabolism. Best Pract. Res. Clin. Endocrinol. Metab..

[53] Deveaux V., Cadoudal T., Ichigotani Y., Teixeira-Clerc F., Louvet A., Manin S., Nhieu J.T.-V., Belot M.P., Zimmer A., Even P. (2009). Cannabinoid CB_2_ receptor potentiates obesity-associated inflammation, insulin resistance and hepatic steatosis. PLoS ONE.

[54] Gatta-Cherifi B., Cota D. (2016). New insights on the role of the endocannabinoid system in the regulation of energy balance. Int. J. Obes..

[55] Lamia K.A., Storch K.-F., Weitz C.J. (2008). Physiological significance of a peripheral tissue circadian clock. Proc. Natl. Acad. Sci. USA.

[56] Fukuda H., Iritani N. (1991). Diurnal variations of lipogenic enzyme mRNA quantities in rat liver. Biochim. Biophys. Acta.

[57] Zardoya R., Diez A., Serradilla M.C., Madrid J.A., Bautista J.M., Garrido-Pertierra A. (1994). Lipogenic activities in rat liver are subjected to circadian rhythms. Rev. Esp. Fisiol..

[58] Pascual A.C., Gaveglio V.L., Giusto N.M., Pasquaré S.J. (2013). Aging modifies the enzymatic activities involved in 2-arachidonoylglycerol metabolism. BioFactors.

[59] Pascual A.C., Martín-Moreno A.M., Giusto N.M., de Ceballos M.L., Pasquaré S.J. (2014). Normal aging in rats and pathological aging in human Alzheimer’s disease decrease FAAH activity: Modulation by cannabinoid agonists. Exp. Gerontol..

[60] Blüher M., Engeli S., Klöting N., Berndt J., Fasshauer M., Bátkai S., Pacher P., Schön M.R., Jordan J., Stumvoll M. (2006). Dysregulation of the peripheral and adipose tissue endocannabinoid system in human abdominal obesity. Diabetes.

[61] Díaz-Asensio C., Setién R., Echevarría E., Casis L., Casis E., Garrido A., Casis O. (2008). Type 1 diabetes alters brain cannabinoid receptor expression and phosphorylation status in rats. Horm. Metab. Res..

[62] You Y., Ren T., Zhang S., Shirima G.G., Cheng Y., Liu X. (2015). Hypoglycemic effects of *Zanthoxylum* alkylamides by enhancing glucose metabolism and ameliorating pancreatic dysfunction in streptozotocin-induced diabetic rats. Food Funct..

[63] Mallat A., Teixeira-Clerc F., Lotersztajn S. (2013). Cannabinoid signaling and liver therapeutics. J. Hepatol..

[64] Gruden G., Barutta F., Kunos G., Pacher P. (2016). Role of the endocannabinoid system in diabetes and diabetic complications. Br. J. Pharmacol..

[65] Weiss L., Zeira M., Reich S., Har-Noy M., Mechoulam R., Slavin S., Gallily R. (2006). Cannabidiol lowers incidence of diabetes in non-obese diabetic mice. Autoimmunity.

[66] Weiss L., Zeira M., Reich S., Slavin S., Raz I., Mechoulam R., Gallily R. (2008). Cannabidiol arrests onset of autoimmune diabetes in NOD mice. Neuropharmacology.

[67] Burstein S. (2015). Cannabidiol (CBD) and its analogs: A review of their effects on inflammation. Bioorg. Med. Chem..

[68] Pfaffl M.W. (2001). A new mathematical model for relative quantification in real-time RT-PCR. Nucleic Acids Res..

[69] Schmittgen T.D., Livak K.J. (2008). Analyzing real-time PCR data by the comparative C_T_ method. Nat. Protoc..

[70] Balsalobre A., Damiola F., Schibler U. (1998). A serum shock induces circadian gene expression in mammalian tissue culture cells. Cell.

[71] Damiola F., Le Minh N., Preitner N., Kornmann B., Fleury-Olela F., Schibler U. (2000). Restricted feeding uncouples circadian oscillators in peripheral tissues from the central pacemaker in the suprachiasmatic nucleus. Genes Dev..

[72] Song C., Howlett A.C. (1995). Rat brain cannabinoid receptors are N-linked glycosylated proteins. Life Sci..

[73] Fukudome Y., Ohno-Shosaku T., Matsui M., Omori Y., Fukaya M., Tsubokawa H., Taketo M.M., Watanabe M., Manabe T., Kano M. (2004). Two distinct classes of muscarinic action on hippocampal inhibitory synapses: M2-mediated direct suppression and M1/M3-mediated indirect suppression through endocannabinoid signalling. Eur. J. Neurosci..

[74] Kallendrusch S., Hobusch C., Ehrlich A., Ziebell S., Ueda N., Geisslinger G., Koch M., Dehghani F. (2012). Site-specific and time-dependent activation of the endocannabinoid system after transection of long-range projections. PLoS ONE.

