# Supplementary material for: Daytime-Dependent Changes of Cannabinoid Receptor Type 1 and Type 2 Expression in Rat Liver
Source: Int J Mol Sci. 2017 Aug 24;18(9):1844. doi: 10.3390/ijms18091844 (PMC5618493; doi:10.3390/ijms18091844)
Supplement: Supplementary file 1 [file ijms-18-01844-s001.pdf]

Supplementary Materials: Daytime-Dependent Changes of Cannabinoid Receptor Type 1 and Type 2 Expression in Rat Liver

Ivonne Bazwinsky-Wutschke, Alexander Zipprich and Faramarz Dehghani

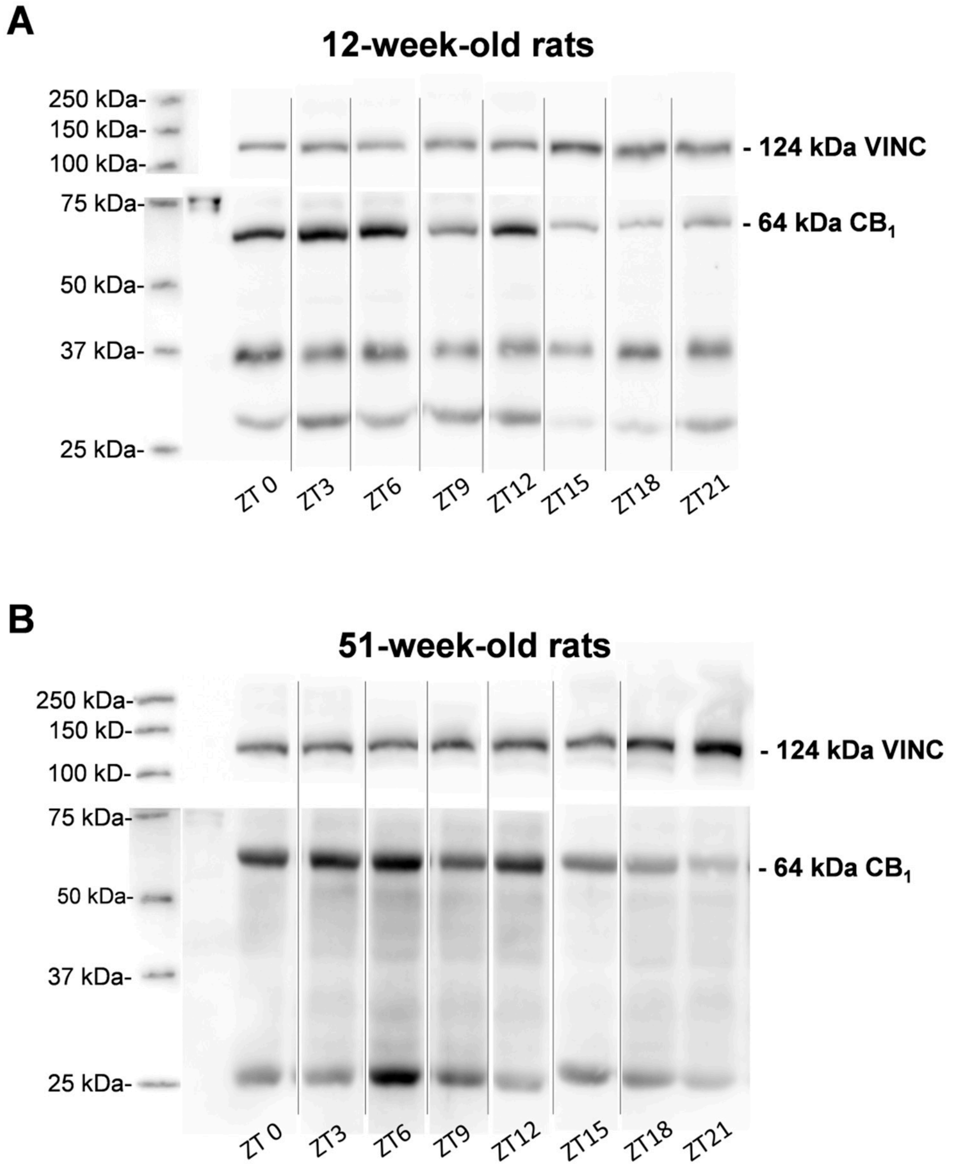

Figure S1. Representative Western blots of rat liver tissue demonstrating diurnal expression pattern of CB_1 (64 kDa) in 12- (**A**) and 51-week-old (**B**) rats at each Zeitgeber Time (ZT). Vinculin (VINC) is the standard for normalization.

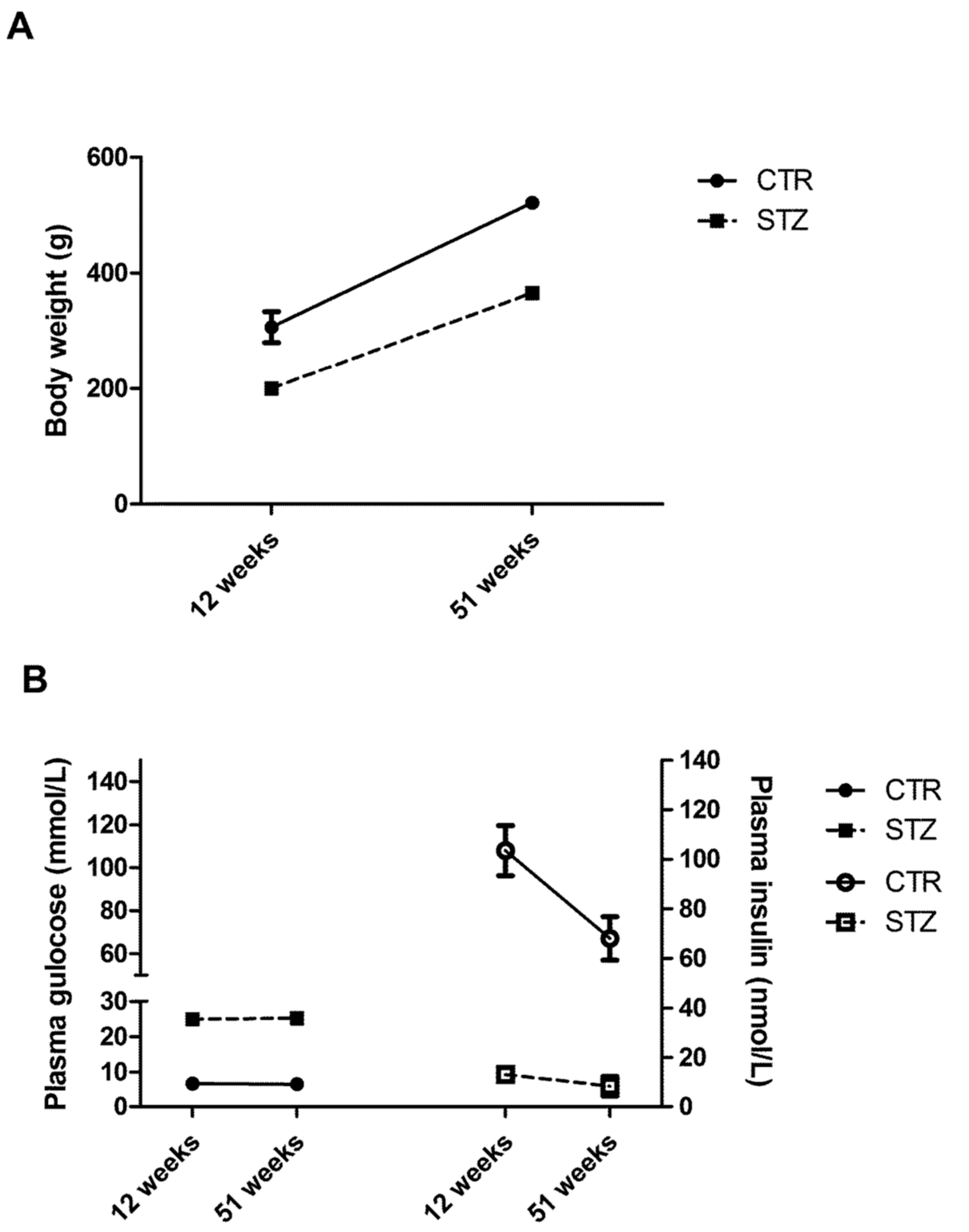

Figure S2. (**A**) Body weight is reduced in streptozotocin (STZ) treated-rats ($p < 0.001$) compared to Wistar rats (CTR). The body weight increases during aging in rats ($p < 0.001$). (**B**) Plasma glucose is significantly increased in STZ rats compared to the corresponding normoglycemic control group of Wistar rats ($p < 0.001$). The plasma insulin levels are very low in STZ rats ($p < 0.001$). Insulin is decreased in 51-week-old compared to12-week-old Wistar rats ($p < 0.01$).
